# Integrated Analysis of DNA Methylation and RNA Transcriptome during *In Vitro* Differentiation of Human Pluripotent Stem Cells into Retinal Pigment Epithelial Cells

**DOI:** 10.1371/journal.pone.0091416

**Published:** 2014-03-17

**Authors:** Zhenshan Liu, Rongfeng Jiang, Songtao Yuan, Na Wang, Yun Feng, Ganlu Hu, Xianmin Zhu, Kevin Huang, Jieliang Ma, Guotong Xu, Qinghuai Liu, Zhigang Xue, Guoping Fan

**Affiliations:** 1 Department of Regenerative Medicine, Translational Center for Stem Cell Research, Tongji Hospital, Tongji University School of Medicine, Shanghai, China; 2 Department of Human Genetics, David Geffen School of Medicine, University of California Los Angeles, Los Angeles, California, United States of America; 3 Department of Ophthalmology, The First Affiliated Hospital of Nanjing Medical University, Nanjing, Jiangsu, China; 4 Tongji Eye Institute and Department of Regenerative Medicine, Tongji University School of Medicine, Shanghai, China; 5 Advanced Institute of Translational Medicine, School of Life Sciences and Technology, Tongji University, Shanghai, China; 6 Suzhou Institute of Tongji University, Suzhou, Jiangsu, China; Wellcome Trust Centre for Stem Cell Research, United Kingdom

## Abstract

Using the paradigm of *in vitro* differentiation of hESCs/iPSCs into retinal pigment epithelial (RPE) cells, we have recently profiled mRNA and miRNA transcriptomes to define a set of RPE mRNA and miRNA signature genes implicated in directed RPE differentiation. In this study, in order to understand the role of DNA methylation in RPE differentiation, we profiled genome-scale DNA methylation patterns using the method of reduced representation bisulfite sequencing (RRBS). We found dynamic waves of *de novo* methylation and demethylation in four stages of RPE differentiation. Integrated analysis of DNA methylation and RPE transcriptomes revealed a reverse-correlation between levels of DNA methylation and expression of a subset of miRNA and mRNA genes that are important for RPE differentiation and function. Gene Ontology (GO) analysis suggested that genes undergoing dynamic methylation changes were related to RPE differentiation and maturation. We further compared methylation patterns among human ESC- and iPSC-derived RPE as well as primary fetal RPE (fRPE) cells, and discovered that specific DNA methylation pattern is useful to classify each of the three types of RPE cells. Our results demonstrate that DNA methylation may serve as biomarkers to characterize the cell differentiation process during the conversion of human pluripotent stem cells into functional RPE cells.

## Introduction

DNA methylation is an important epigenetic modification involved in numerous cellular processes, including embryonic development [1]–[3], genomic imprinting [4], [5], X-chromosome inactivation [6], [7], and chromosome stability [8]. During development, DNA methylation plays an important role in epigenetic programming by silencing stem cell-specific genes and activating differentiation-associated genes [9], [10]. Recent studies using high-throughput sequencing technologies have mapped the genome-wide DNA methylation changes at the single nucleotide resolution. These studies have uncovered that DNA methylation contributes to cellular lineage commitment *in vitro* [11]–[13] and *in vivo* [14]–[17].

The retinal pigment epithelium (RPE) is a monolayer of terminally differentiated pigmented cells between the neural retina and choroid. RPE is involved in the formation of the blood-retinal barrier, absorption of stray light, supplying of nutrients to the neural retina, regeneration of visual pigment, as well as the uptake and recycling of the outer segments of photoreceptors. We and others have generated functional RPE through *in vitro* differentiation of both human embryonic stem cells (hESCs) and induced pluripotent stem cells (hiPSCs) [18]–[24]. Furthermore, RPE derived from hESCs and hiPSCs can be injected into the subretinal space where normal RPE resides and restore visual function in the retinal dystrophy rat model [23], [25]. To understand the gene regulation of key genes during *in vitro* differentiation of hESCs/iPSCs into RPE, we had previously identified RPE mRNA signature genes [20] and demonstrated that RPE-specific miRNAs were associated with the RPE differentiation and maturation of RPE *in vitro* [24].

In this study, we mapped genome-scale DNA methylation at single-base resolution in fetal RPE as well as hESC- and hiPSC-derived RPE (hESC-RPE and hiPSC-RPE) and found cell-type specific DNA methylation pattern. Furthermore, we correlated promoter DNA methylation with both mRNA and miRNA gene expression during *in vitro* RPE differentiation from pluripotent hESCs.

## Results

### Profiling genome-scale DNA methylation patterns during the differentiation of human stem cells into RPE cells

We have derived functional RPE cells from multiple lines of human pluripotent stem cells, including a total of thirteen lines of hESCs and iPSCs through *in vitro* differentiation over the course of three to six months [20], [24] (data not shown). In our observations, we found that both H9 and UCLA4 hESCs, as well as hiPSC2 and HDF2 iPSCs are representative of all hESCs and iPSCs in the RPE differentiation time course [20]. Moreover, the cellular biological profile of both hESC-derived RPE and iPSC-derived RPE cells have been well characterized including RPE marker expression and RPE functional phagocytosis assays [20], [24] (data not shown). Using H9 hESCs as a model, we characterized its DNA methylation profiles during directed RPE differentiation and cross-referenced methylation profiles with mRNA and miRNA expression profiles. We also profiled DNA methylation in two well characterized primary fetal RPE cells and defined a cell-type specific DNA methylation pattern among fetal RPE and hESC- and hiPSC-RPE [20], [24]. We first isolated genomic DNA from fetal RPE cells, and from hESCs and hiPSCs at four distinct stages during *in-vitro* differentiation into RPE cells respectively, *i.e.*,1) pluripotent stem cells (H9 and UCLA4 hESCs as well as hiPSC2 and HDF2 iPSCs); 2) 15 days partially differentiated ES cells (H9 hESC); 3) early pigmented clusters after 30-day *in vitro* differentiation (H9 hESC); and 4) functional RPE (3–6 months in culture, H9 and UCLA4 hESCs as well as hiPSC2 and HDF2 iPSCs). We then performed DNA methylation mapping by RRBS, which is a robust, quantitative, and effective approach to map global DNA methylation. Our RRBS analyses covered on average of about 1 million individual CpGs throughout the human genome (Table S1), including those found within over ten thousand unique gene promoters.

**Figure 1:**
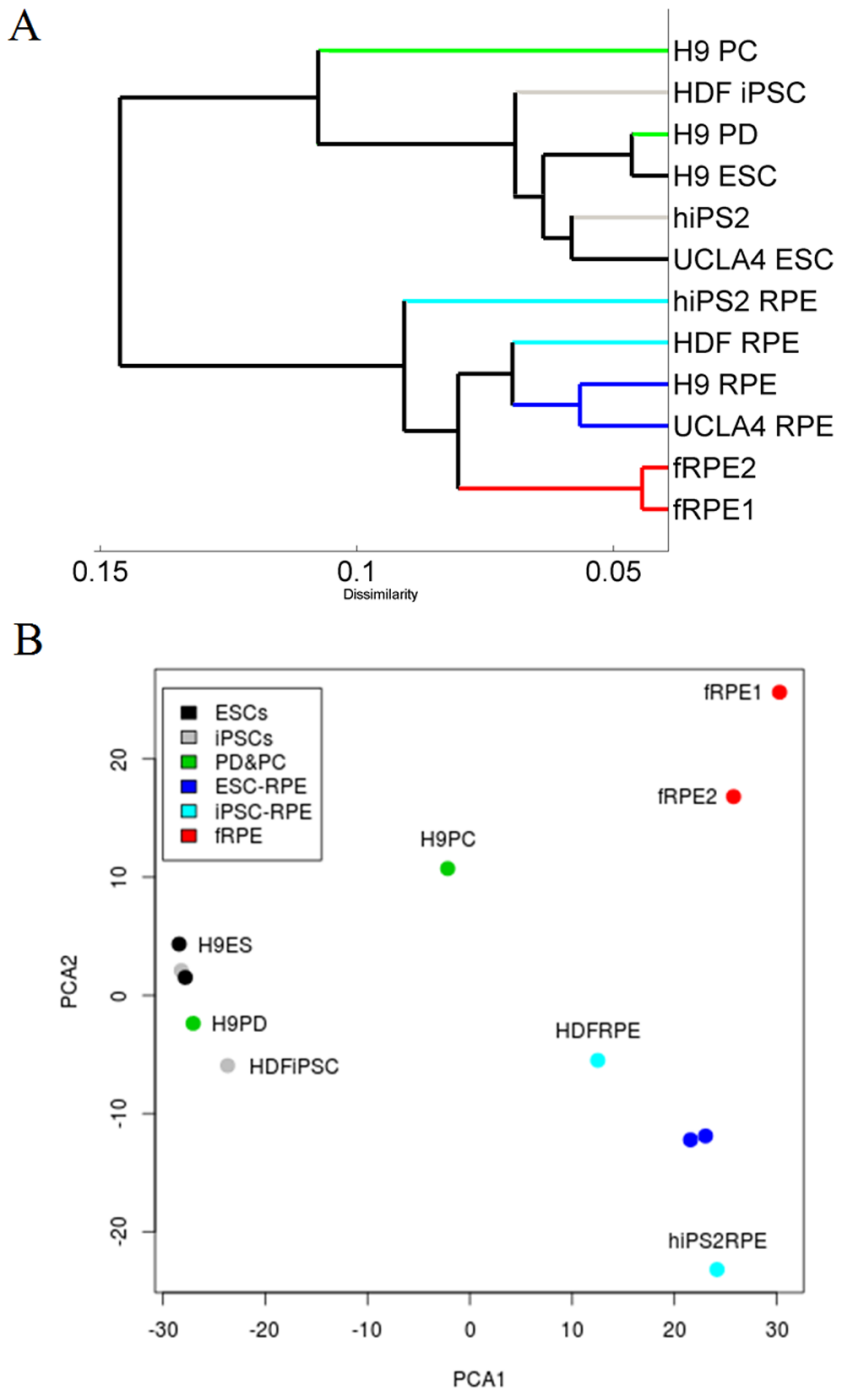
Different cell types exhibit distinctive global DNA methylation patterns. DNA methylation profiles of different cell types were clustered using either unbiased hierarchical clustering or principal component analysis (PCA) based on the DNA methylation levels of all shared CGs in all cell lines analyzed. A) Hierarchal clustering using Pearson correlation distance between methylation levels of 535,376 shared CGs. Defined cell types show a high overall similarity in the methylation patterns and thus cluster together. B) 2D-biplot of the first two principal components. For this analysis, we report the average principal component scores after randomly sampling of 100,000 shared CGs over 200 iterations.

To assess whether DNA methylation patterns distinguish cell types, we performed hierarchical clustering and principal component analysis (PCA) based on genome-wide CG methylation levels (Figure 1). Both clustering methods revealed that terminally differentiated cells clustered distinctly away from immature cell types, such as ESCs and iPSCs and partially differentiated cells. Furthermore, we observed ESC-derived RPE (ESC-RPE) were more similar to each other than iPSC-derived RPE (iPSC-RPE). However, both ESC-RPE and iPSC-RPE were distinctly different to fetal RPE. Overall, identical cell types clustered tightly together, suggesting that each cell-type exhibits a well-defined DNA methylation pattern. These observations were reminiscent of what we have observed with mRNA and miRNA expression patterns [20], [24], suggesting that DNA methylation patterns are as equally useful as gene expression profiles for identifying different cell types during RPE differentiation.

We next examined the DNA methylation dynamics at four distinct stages of RPE differentiation, taking the well-studied H9 ESCs as an example (Figure S1A). We found that the level of CpG methylation was initially increased upon partial stem cell differentiation, then decreased during lineage-specification, and then increased again upon RPE maturation (Figure S1B). In addition to the difference in overall methylation, we also analyzed the distribution of the CpG methylation levels in the four RPE differentiation stages (Figure S2). These findings indicate that both *de novo* methylation and demethylation dynamically take place during RPE differentiation.

From undifferentiated hESCs (Stage I) to partially differentiated ESCs (PD, Stage II), the first wave of methylation changes was dominated by DNA hypermethylation. In contrast, the transition from Stage II partially differentiated ES cells to Stage III pigmented clusters showed more hypomethylation than hypermethylation (Figure S1B). To understand which genomic elements undergo DNA methylation changes during RPE differentiation, we next analyzed the distribution of hyper- and hypo-methylated CpG sites between two adjacent stages. The results showed that while repeat elements [long interspersed nuclear element (LINE), long terminal repeat (LTR), and satellite repeats] and gene bodies did not exhibit much difference, a subset of CpG islands (CGI) and short interspersed nuclear elements (SINEs) showed differential methylation during RPE differentiation (Figure S1C and D, Figure S3). This suggests that different genomic regions would be subject to either increase, or decrease, or no change in DNA methylation.

### Changes in promoter methylation during RPE differentiation

**Figure 2:**
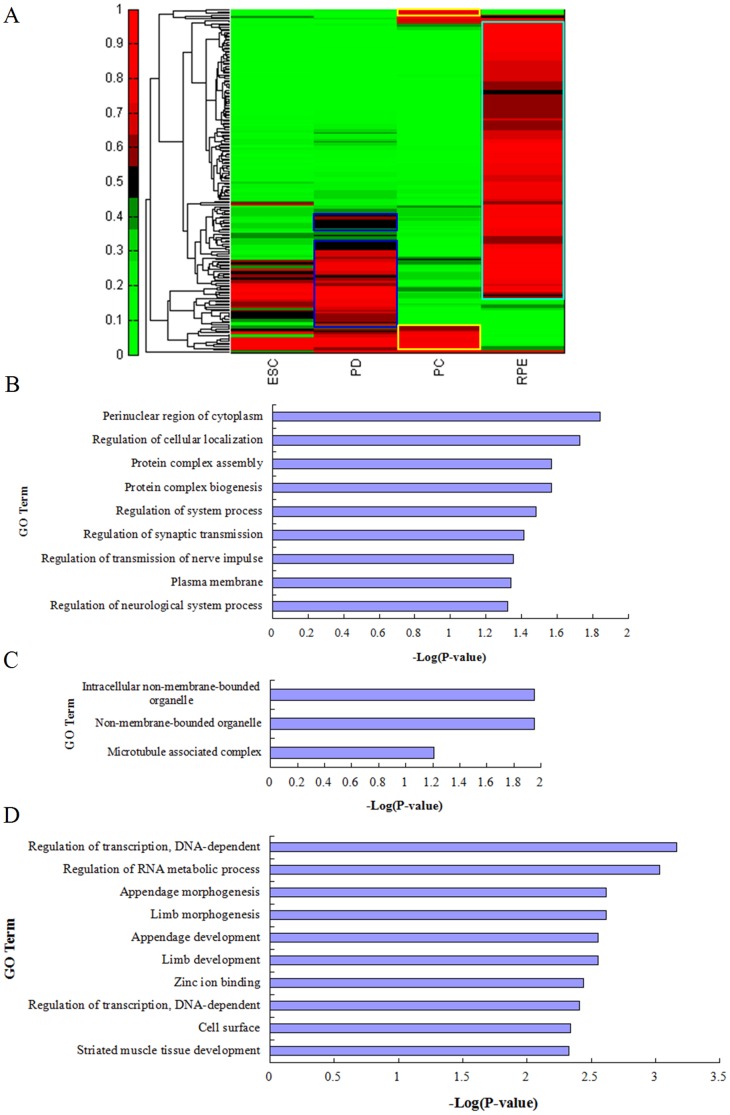
Analysis of differentially methylated genes during RPE differentiation from human H9 ESCs. (A) Heatmap analysis of promoter methylation of differentially methylated genes during RPE differentiation. ESC is undifferentiated H9 hESCs, PD: partial differentiated H9 cells, PC: pigmented cluster. RPE: H9 ESC-RPE. (B, C, D) GO analysis of differentially methylated genes during RPE differentiation. Bar graphs showing significance of enrichment terms for sets of demethylated genes from PD into PC cells (B, as indicated by the two blue boxes in figure 2A) and during the course of RPE maturation from PC to RPE (C, the yellow box in figure 2A), and remethylated genes in mature RPE (D, the two light blue boxes in figure 2A) as listed in Table S2, S3, S4. P-values<0.05.

Recent studies highlight that DNA methylation patterns change significantly from pluripotent stem cells or multipotent progenitors to lineage-committed cells [11], [26], [27]. Consistently, methylation in a subset of genes is dynamically regulated during RPE differentiation (Figure 2A, File S1). In the transition stage between Stage II PD cells and Stage III PC cells, demethylated genes were enriched in protein complex biogenesis and neurological system process [genes in blue box in Figure 2A and gene ontology (GO) terms in Figure 2B, Table S2]. In contrast, a subset of genes associated with non-membrane-bounded organelle and transcription factor activity exhibits increased methylation (data not shown). Interestingly the genes associated with non-membrane-bounded organelle were demethylated during RPE maturation (Stage III to Stage IV, yellow box in Figure 2A and GO terms in Figure 2C, Table S3). GO annotations for all the re-methylated genes showed significant enrichment of genes in the developmental processes and transcriptional regulation in mature RPE cells (light blue box in Figure 2A and GO term in Figure 2D, Table S4). This result implied that DNA methylation is associated with the silencing of developmental genes such as LAMC2, ALX3, and SALL4 [28] in the terminally differentiated RPE cells (Figure S4). Overall, our analysis indicates that stage-specific DNA methylation patterns can reveal developmentally regulated RPE-differentiation genes and classify cell differentiation process.

### DNA methylation and miRNA expression changes during RPE differentiation

**Figure 3:**
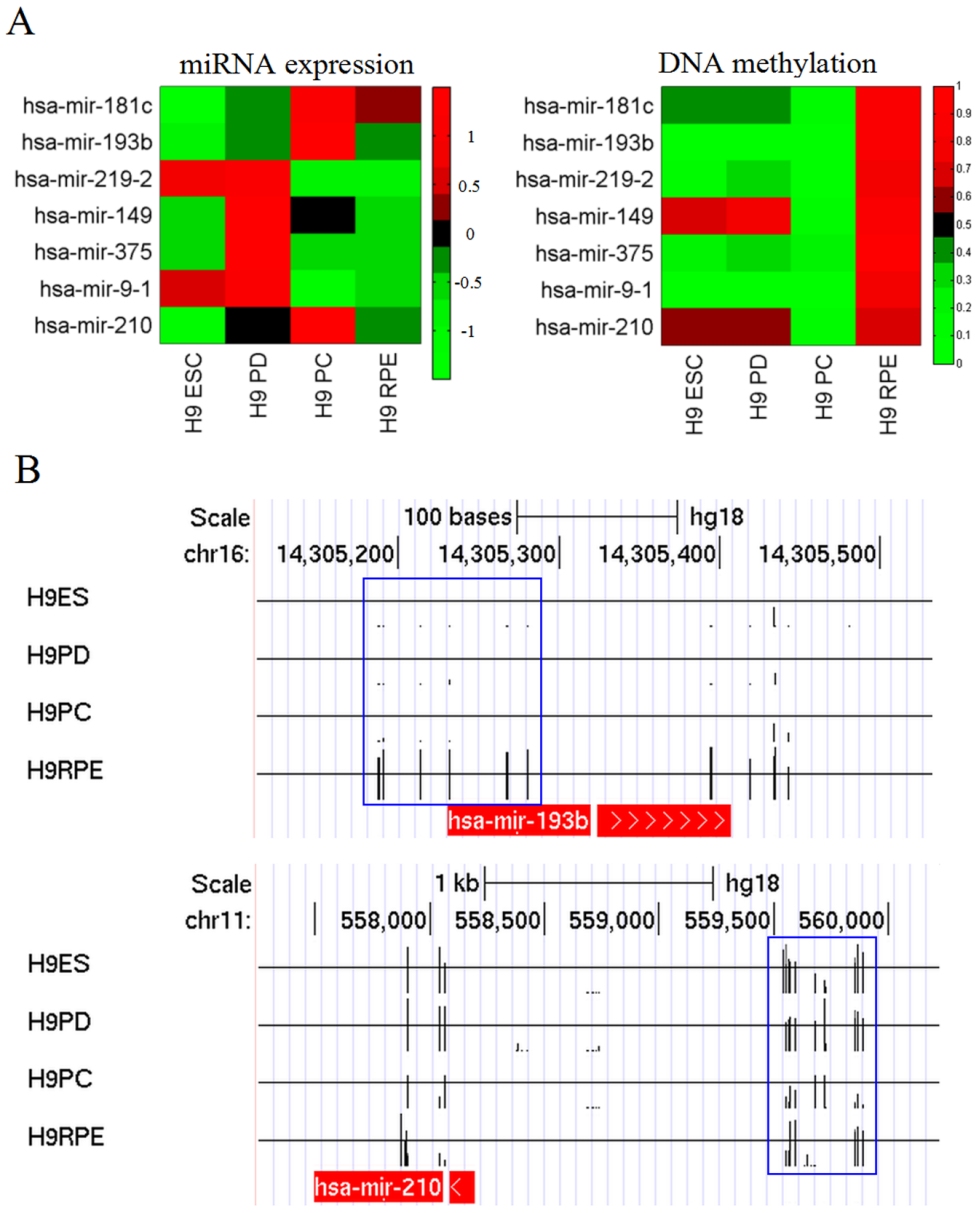
DNA methylation and miRNA expression changes during RPE differentiation. (A) Heatmap analysis of differentially expressed miRNAs during the course of differentiating H9 ESCs. miRNA expression levels were standardized by the mean level in all stages, whereas DNA methylation levels not standardized. DNA methylation values were the average levels between −1000 bp upstream and +500 bp downstream of the TSS. (B) Genome browser views of DNA methylation patterns found in hsa-mir-193b and hsa-mir-210 loci during the course of RPE differentiation.

MicroRNAs are small non-coding RNAs which are expressed in a tissue-specific manner and play important roles in cell proliferation and differentiation. DNA methylation-mediated downregulation of miRNA gene expression has been observed in various types of cancers [29]. Since we have recently profiled the miRNA expression pattern during RPE differentiation [24], we further analyzed the data to determine any correlation between DNA methylation and miRNA expression. We analyzed the expression and DNA methylation levels in a total of 419 miRNAs, and selected 216 highly and 92 lowly methylated miRNAs based on H9 RPE methylation levels (methylation levels>0.8 were defined as high, and methylation levels<0.2 were defined as low, see Figure S5). In mature RPE, we found a negative correlation between DNA methylation and miRNA expression for hsa-mir-193b and hsa-mir-210 clusters in the four different cell stages (Figure 3), indicating that DNA methylation is associated with the silencing of these two miRNAs. However, we also found 18 cases in which DNA methylation and miRNA expression were not anti-correlated (e.g., has-mir-181c in Figure 3A), suggesting a methylation-independent mechanism for the expression regulation of these microRNAs.

### Most RPE mRNA signature genes are demethylated at proximal promoter regions in both hESCs and RPE cells

**Figure 4:**
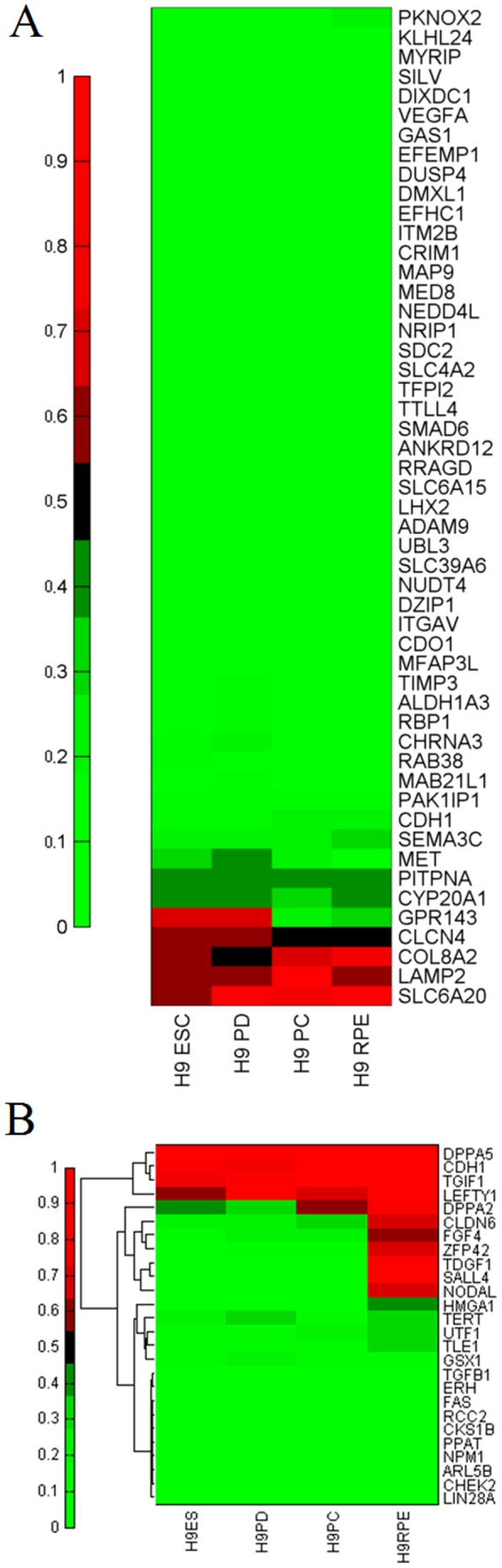
DNA methylation profiles of RPE and ESC signature genes during the course of RPE differentiation. Heatmap showing the average promoter methylation levels of (A) RPE and (B) ESC mRNA signature genes during the conversion of stem cells into functional RPE.

DNA methylation is linked to gene silencing and considered to be an important mechanism in the regulation of mRNA transcription. Previously we have identified a set of 87 RPE signature genes in fetal and stem cell-derived RPE [20]. To examine the relationship between DNA demethylation and gene expression of these RPE mRNA signature genes, we determined the DNA methylation levels of promoters in pluripotent stem cells and RPE. Surprisingly, among the 46 RPE mRNA signature genes analyzed by RRBS method, we found most of these genes maintained hypomethylation on their proximal promoters in both stem cells and RPE cells. Our observations suggested that either: 1) gene coverage via RRBS is incomplete, thus inadequate to predict the status of other signature genes; or 2) demethylated promoters are permissive to expression, but only activated via other regulatory mechanisms in mature RPE cells. However, two RPE signature genes, G protein-coupled receptor 143 (GPR143) and chloride channel (CLCN4) exhibited promoter demethylation coupled with gene activation, implicating that DNA demethylation contributes to the activation of these two RPE signature genes from the transition of stem/progenitor cells to pigmented cluster/RPE cells (Figure 4A). Next, we analyzed the DNA methylation of 26 ESC-specific genes during RPE differentiation [30]–[32]. We found that the methylation levels significantly increase in approximately 30% of genes (e.g. DPPA2, TDGF1 and SALL4) in differentiated RPE cells, consistent with the function of DNA methylation in silencing pluripotency genes in differentiated somatic cells (Figure 4B).

### Genes related to cell adhesion and extracellular matrix are hypomethylated in fetal RPE

**Figure 5:**
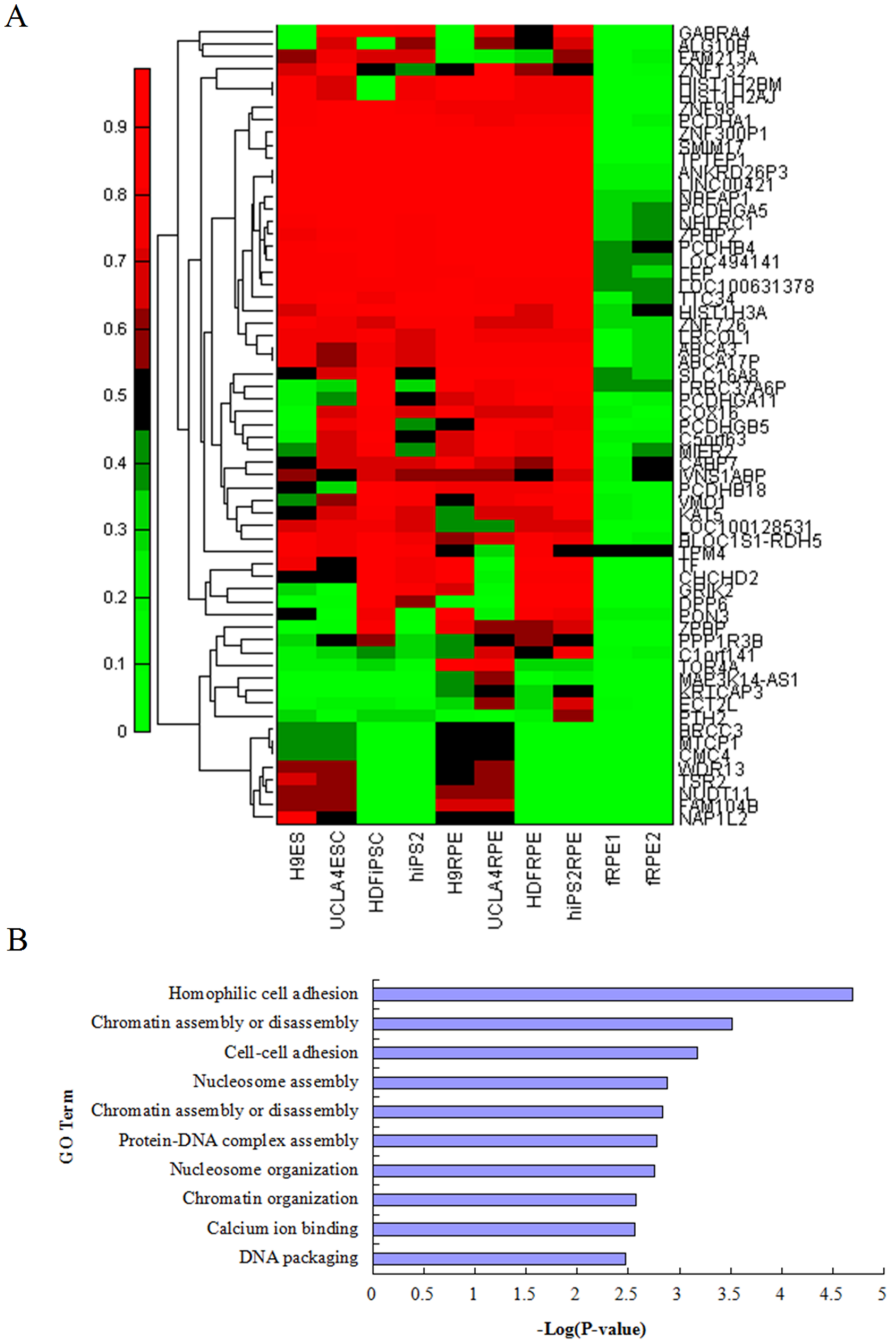
Differential DNA methylation in hESC-/hiPSC-RPEs and fRPEs. (A) Heatmap analysis of differential methylated genes in hESC/hiPSC-RPEs and fRPEs. (B) GO analysis of fRPE-specific demethylated genes.

**Figure 6:**
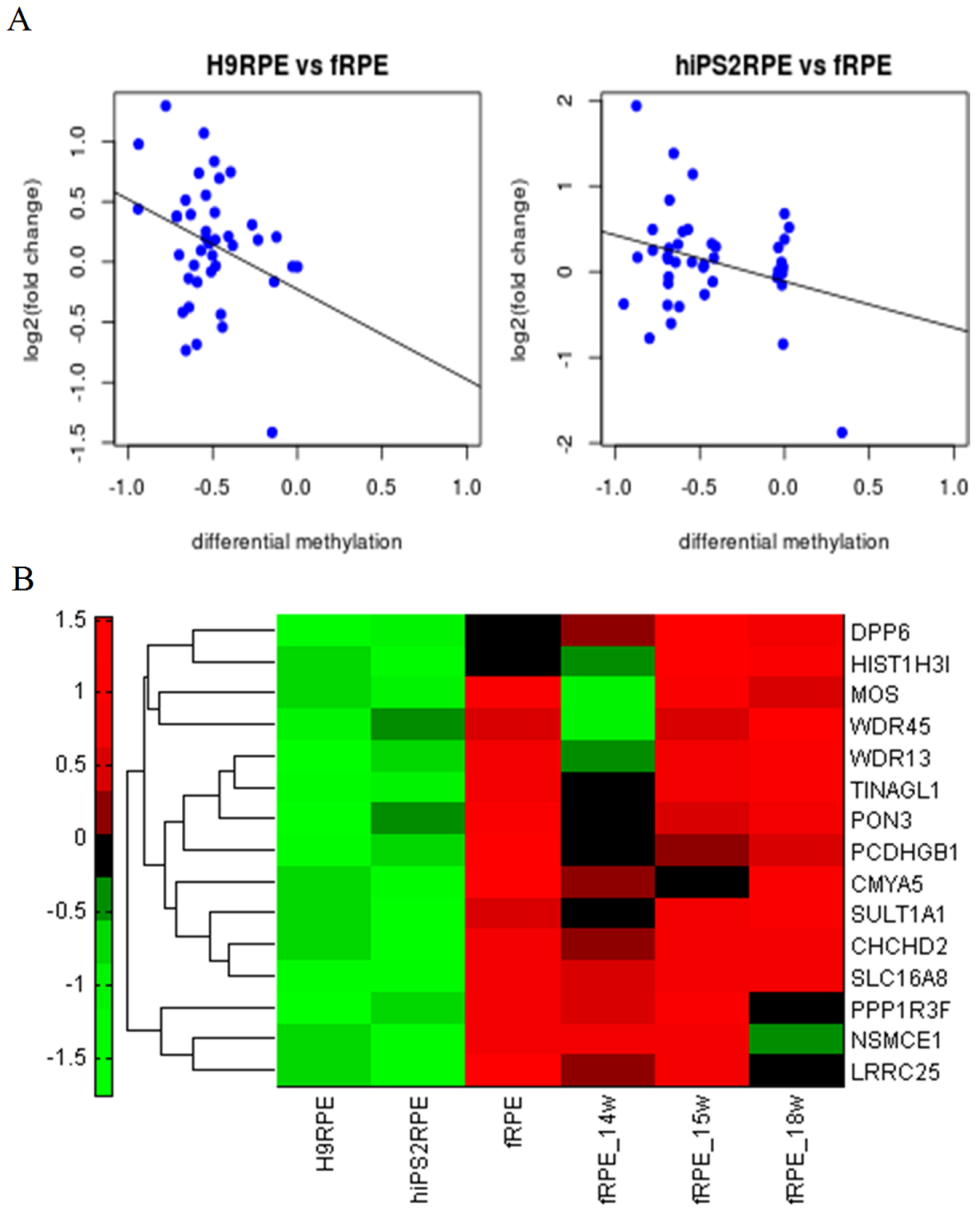
The correlation of gene expression and DNA methylation in hESC-/hiPSC-RPEs and fRPEs. (A) Scatterplots of gene expression fold change vs DNA methylation change in hESC- and hiPSC- RPEs vs. fRPEs. The correlation coefficients were −0.306 (p-value = 0.051) and −0.288 (p-value = 0.067), respectively. (B) Gene expression heatmap analysis of selected genes that are methylated in H9 ESC-RPE, but not in fetal RPE.

Recent studies found that hESC-RPEs and iPSC-RPEs expressed essential RPE markers and could rescue visual function in animal models, and have the potential to treat a wide range of retinal diseases [18], [19], [23], [33]. Our previous study found that despite morphological and functional similarities, gene expression of stem-cell-derived RPE cells were moderately different from normal fRPE cells, and that fRPE-specific genes were important for eye development [20]. To examine potential difference in DNA methylation among stem-cell-derived RPE cells and fetal RPE, we performed clustering analysis of specific regions that exhibit variations in DNA methylation (Figure 5A). We found that fetal RPE exhibited a set of demethylated genes that were highly methylated in both hESC-RPE and hiPSC-RPE. GO analysis revealed that these genes were associated with cell adhesion and ion binding (Figure 5B, Table S5). These differentially methylated genes were modestly inversely-correlated with gene expression (Figure 6). We also found that hESC-RPEs and iPSC-RPEs had slightly different DNA methylation profiles from each other (e.g., 20 differentially methylated genes as in Figure 5A). GO analysis revealed that these genes were associated with peptidase activity (data not shown). These results demonstrate that significant differences in DNA methylation profiles exist among hESC-RPE, iPSC-RPE, and fRPE. Many of these CpG methylation patterns were already established in undifferentiated hESCs and iPSCs (Figure 5A). Furthermore, some differential methylation between hESC-RPE and iPSC-RPE are reflected in previous differential methylation found between undifferentiated hESCs and iPSCs (the bottom quarter of Figure 5A, also see reference [34]).

## Discussion

Gene expression profiles have been shown to accurately reflect cell type and differentiation stage [16], [35], [36]. In this study, we found that four stages of RPE differentiation can be distinguished through a unique subset of DNA methylation patterns. Although previous studies have shown that iPSCs and ESCs are similar in terms of transcription program, chromatin modification profiles [37]–[42], and global chromatin configuration [37], [43], [44], differences in epigenomics [34], [45]–[47] and differentiation potential between iPSCs and ESCs [48]–[50] have been previously reported. In this study, by establishing high-resolution genomic maps of DNA methylation for stem-cell-derived RPE and human fRPE cells, we revealed the distinct differences in DNA methylation among hESC-RPE, iPSC-RPE, and fRPE. Our findings also support the idea that hESCs and iPSCs may have inherent differences in DNA methylation patterns [34], [41], [42], [45].

Stem cell differentiation is a process characterized by the progressive loss of developmental potential and gain in functional specialization, and DNA methylation dynamics has previously been investigated during cell differentiation [11]–[14]. Our results indicate that both demethylation and *de novo* methylation take place during directed RPE differentiation from hESCs and iPSCs, consistent with previous findings. It is known that the DNA methylation change takes place during extended passages of stem cells *in vitro* [51], [52]. Furthermore, different culture conditions can affect DNA methylation patterns *in vitro* [53]–[55]. In this study, we found that the methylation differences between hESC and iPSC lines are relatively small when compared to the differences found in differentiated hESC-RPE and hiPSC-RPE. This result implicates that cell differentiation may amplify the methylation differences in hESCs and hiPSCs in their somatic derivatives.

Previous studies have revealed that promoter DNA methylation has an important effect on regulating cell type specific genes, which ultimately contributes to both cell physiology and morphology [56], [57]. Ji *et al.* found that differential DNA methylation is more strongly correlated with gene expression at CpG island shores than CpG islands [15]. Moreover, intragenic methylation could also have an important role in regulating cell context-specific alternative promoters in gene bodies [58]. However, other studies argued that DNA methylaiton might only exert a minor influence on the regulation of tissue-specific gene expression [59]. In this study, we found a negative correlation between promoter DNA methylation and gene expression for some genes (e.g., CHCHD2, SLC16A8, hsa-mir-193b and hsa-mir-210), which is consistent with the hypothesis that promoter methylation represses gene expression. Nevertheless, for many RPE signature genes, DNA methylation and gene expression did not show strong negative correlation during RPE differentiation. One explanation for this observation is that our RRBS assay is limited in genomic coverage and fails to reveal differential methylation patterns. Alternatively, we can not exclude the fact that other regulatory mechanism(s) independent of DNA methylation is crucial for regulating RPE-specific genes. Future study with comprehensive profiling of genome-wide DNA methylation/histone modifications and genetic manipulation of DNA methylation pathway in RPE cells may shed additional insights into the relationship between methylation and RPE gene regulation during cell differentiation.

Dysfunction, degeneration, and loss of RPE cells are prominent features of Best disease, subtypes of retinitis pigmentosa (RP), and age-related macular degeneration (AMD). Because current treatments for these diseases are severely limited, stem cell-based replacement therapy involving RPE transplantation holds tremendous promise. Human pluripotent stem cells (hESC and hiPSC) may serve as an unlimited donor source of RPE cells for transplantation. Previous studies on differentiating RPE cells from stem cells demonstrated that stem-cell-derived RPE cells had molecular characteristics similar to primary RPE cells [18], [19], [33]. Moreover, the transplantation of stem-cell-derived RPE could partially restore visual function in the retinal dystrophy rat model [23], [33], [60]. To assess the potential of stem cell-derived RPE for cell replacement therapy, Sugino *et al* compared the attachment and survival of hESC-RPE of different degrees of pigmentation on Bruch's Membrane (BM) with cultured human fRPE. They found that hESC-RPE showed impaired initial attachment, and cell behavior and protein secretion were markedly dissimilar [61]. Notably, in the present study, we found that an obvious difference in DNA methylation between fRPE and stem cell-derived RPE exists for genes involved in cell adhesion and ion transport. It will be of great interest to understand whether differential methylation in fetal and stem cell-derived RPE leads to any functional alteration in RPE cell adhesion in future cell transplantation studies.

In conclusion, our results indicate that DNA methylation patterns are dynamically regulated during RPE differentiation, and there is an obvious variability among human ESC- and iPSC- RPE as well as primary fRPE cells. These observations demonstrate that DNA methylation accurately reflects cellular identity and distinguishes different stages during RPE differentiation. Integrated analysis of DNA methylation and RPE transcriptome revealed a reverse-correlation between levels of DNA methylation and expression of a small subset of miRNA and mRNA-coding genes that are critical for RPE differentiation and maturation, suggesting that DNA methylation plays an important role in directed RPE differentiation from pluripotent stem cells.

## Materials and Methods

### Differentiation of hESC/hiPSC-RPE

The human embryonic stem cell lines H9 and UCLA4, and induced pluripotent stem cell lines hiPSC2 and HDF iPSC were obtained from the UCLA Stem Cell Core [20]. Fetal RPE cell lines fRPE1 and fRPE2, and mouse embryonic feeder cells were generated in Dr. Guoping Fan's lab at UCLA [20]. Pluripotent stem cells (hESC and iPSCs) were plated onto gamma-rays irradiation mouse embryonic feeder cells with DMEM/F12 culture medium containing 20% Knock-Out Serum Replacement, 0.1 mM nonessential amino acids, 0.1 mM β-mercaptoethanol and 100 ng/ml zebrafish basic fibroblast growth factor (zfbFGF) on a 6-well plate. Cells were cultured at 37° in 5% CO2 for 6–10 days after which zfbFGF was omitted to facilitate spontaneous cell differentiation.

Pigmented colonies were observed within 4 weeks and allowed to expand for a few weeks, with media changes every 2–3 days. Pigmented cells were enriched by manual dissection using insulin needle followed by seeding on growth factor reduced Matrigel (BD Biosciences, diluted 1∶30) coated plate and transwell membranes.

RPE medium were changed to support pigment cluster expansion [containing α-MEM, 1×N2 supplement (Gibco), 1×Non-essential amino acid solution, 250 mg/ml taurine, 13 ng/ml Triiodothyronin (Sigma-Aldrich, Gillingham, UK), 20 ng/ml Hydrocortisone (Sigma), 2 mM L-glutamine (Invitrogen, Paisley, UK), 1×Penicillin-streptomycin and 10% Hyclone heat-inactivated fetal bovine serum (Thermo Scientific, Northumberland, UK)], which was replaced daily.

### DNA Isolation

Genomic DNA was isolated from all samples by traditional phenol/chloroform method. DNA quality was controlled by agarose gel electrophoresis and quantified by a NanoDrop ND-1000 Spectrometer (PeqLab Biotechnologies, Erlangen, Germany).

### Reduced representation bisulfite sequencing

Reduced representation bisulfite sequencing (RRBS) was performed as described [62]. RRBS reads cover less than 10% of the 28 million CpGs in the human genome [63]. Briefly, 1 µg genomic DNA was digested with the methylation insensitive restriction enzyme MspI (NEB). Ends of each restriction fragment were filled in and a 3′ adenosine was added with Klenow Fragment (3′→5′ exo-minus, NEB). Methylated paired-end Illumina adapters were ligated to the ends of the DNA fragments using T4 DNA Ligase (NEB). Fragments between 100 bp and 400 bp were purified by agarose gel extraction. The purified fragments were treated with sodium bisulfite and then amplified by PCR. The final PCR products were sequenced on Illumina HiSeq2000 machines.

### Statistical analysis and bioinformatics

RefSeq genes (NCBI36/hg18) were downloaded from UCSC Genome Browser (http://genome.ucsc.edu/). A RRBS hg18 genome was generated by *in silico* digestion of the genome, followed by *in silico* bisulfite conversion and size selection (25–350 bp). Regions outside the theoretical size selection range were made unmappable by replacing with Ns.. Reads were then mapped to this RRBS genome using BS-Seeker [64], and methylation calling was performed as previously described [11]. Individual cytosine methylation levels were defined as the number of unconverted cytosine over number of unconverted and converted cytosine [#C/(#T+#C)]. Only cytosines that were covered at least 5 reads were considered for further downstream analysis. Clustering analysis and heatmaps were performed using built-in functions in Matlab. Only CGs that were found in all cell types assayed were considered. For DNA methylation heatmaps, colors represent average methylation levels ranging between 0–100%.

For successive stage changes in CG methylation, we examined the number of shared CGs that changed by >0.5, which are significantly differentially methylated as tested by binomial cumulative distribution function. The shared CGs were classified as hyper- or hypo-methylated if the absolute methylation difference between two stages was ≥0.5. For promoters, we considered only CGs that were −1000 bp to +500 bp of the transcription start site (TSS), and required differential methylation difference of >0.5 and p-value<0.05 as calculated by two-sample Kolmogorov-Smirnov test. Genomic distribution of CGs were performed using annotateBed found in the BEDTools package [65]. GO analysis was performed using DAVID [66].

### Accession IDs

RRBS data reported in this paper have been deposited in the NCBI Gene Expression Omnibus (GEO) database with accession number **GSE43473**.

## Supporting Information

Figure S1 **Dynamic changes in DNA methylation during RPE differentiation.** (A) Bar graph displaying the mean CG methylation level of all assayed CGs shared between the H9 line of RPE (N = 733,672). (B) Bar graph showing the proportion of hyper- and hypo-methylated CpG sites between two adjacent stages during RPE differentiation (differential methylation >50%). (C, D) Metaplot analysis of average CG methylation changes for (C) CpG islands and (D) SINE during RPE differentiation.
(TIF)

Figure S2 **The distribution of CG methylation level during RPE differentiation.**
(TIF)

Figure S3 **The distribution of CG methylation level for (A) LINE, (B) LTR, (C) SINE, (D) Satellite, (E) CGI, (F) Genebody, (G) Exon and (H) Intron during RPE differentiation.**
(TIF)

Figure S4 **Genome browser views of DNA methylation profiles found in LAMC2, ALX3, and SALL4 genes during the course of RPE differentiation.**
(TIF)

Figure S5 **The overview of DNA methylation and expression of miRNAs.** (A, C, E) Boxplots of DNA methylation levels for 419 all, 216 high and 92 low selected miRNAs, respectively. (B, D, F) Boxplots of miRNA expression levels on log scale for 419 all, selected 216 high and 92 low methylated miRNAs.
(TIF)

Table S1 **Summary of RRBS reads and mapping data.**
(DOC)

Table S2 **GO analysis via DAVID software for set of demethylated genes from PD into PC cells.**
(DOC)

Table S3 **GO analysis via DAVID software for set of demethylated genes from PC into RPE cells.**
(DOC)

Table S4 **GO analysis via DAVID software for set of remethylated genes from PC to mature RPE.**
(DOC)

Table S5 **GO analysis via DAVID software for fRPE-specific demethylated genes comparing with hESC-RPEs and iPSC-RPEs.**
(DOC)

File S1 **The gene names and the methylation values for the different samples underlying** **Figure 2A** **.**
(XLS)
