# Supplementary figures and images for: Integrated Analysis of DNA Methylation and RNA Transcriptome during In Vitro Differentiation of Human Pluripotent Stem Cells into Retinal Pigment Epithelial Cells
Source: PLoS One. 2014 Mar 17;9(3):e91416. doi: 10.1371/journal.pone.0091416 (PMC3956675; doi:10.1371/journal.pone.0091416)

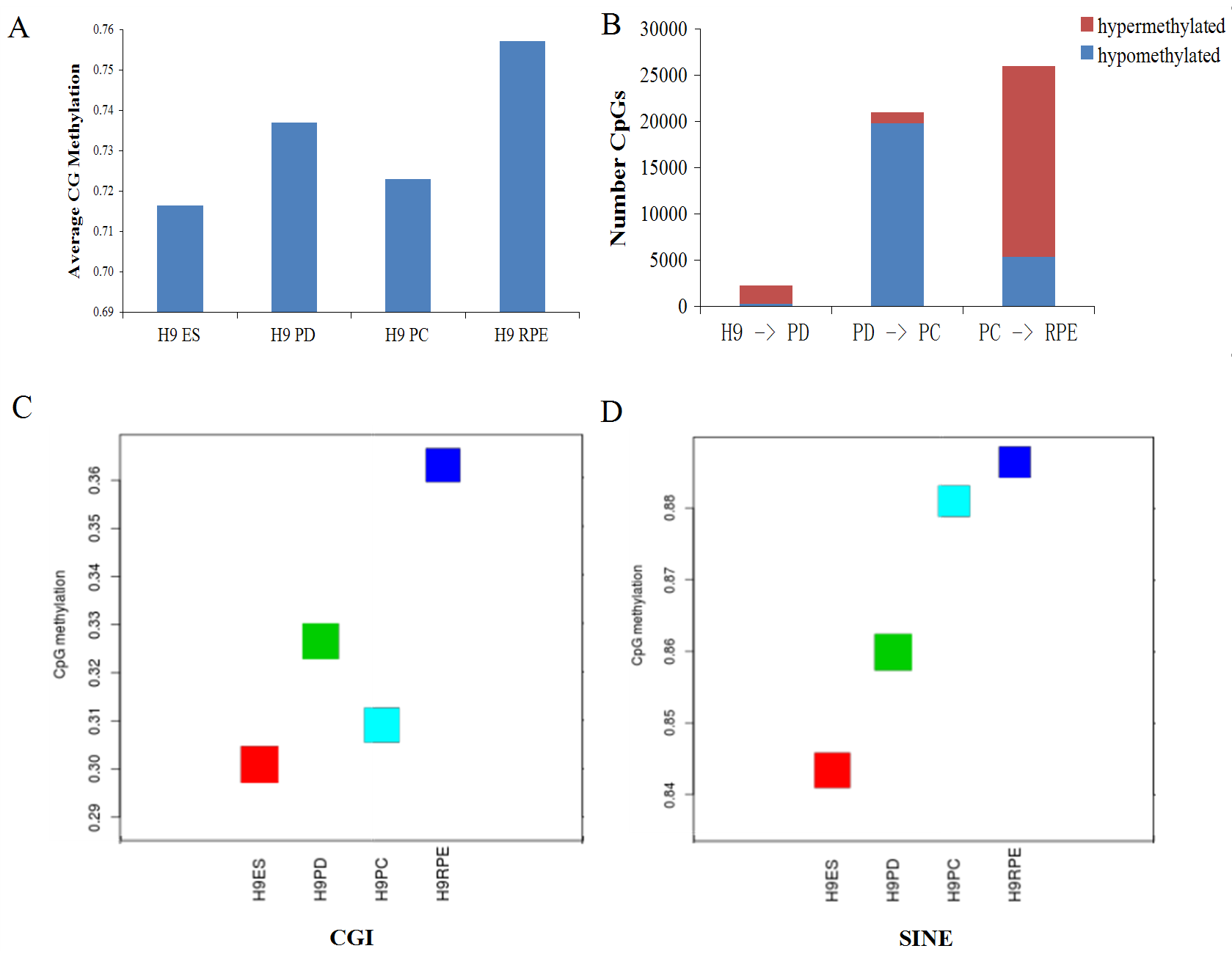

Supplement: Figure S1 — Dynamic changes in DNA methylation during RPE differentiation. (A) Bar graph displaying the mean CG methylation level of all assayed CGs shared between the H9 line of RPE (N = 733,672). (B) Bar graph showing the proportion of hyper- and hypo-methylated CpG sites between two adjacent stages during RPE differentiation (differential methylation >50%). (C, D) Metaplot analysis of average CG methylation changes for (C) CpG islands and (D) SINE during RPE differentiation. (TIF) [file pone.0091416.s001.tif]

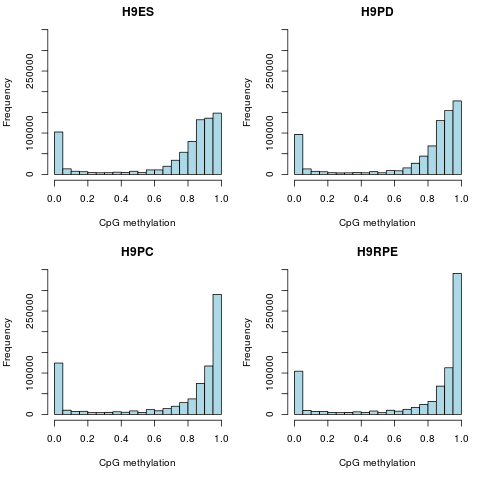

Supplement: Figure S2 — The distribution of CG methylation level during RPE differentiation. (TIF) [file pone.0091416.s002.tif]

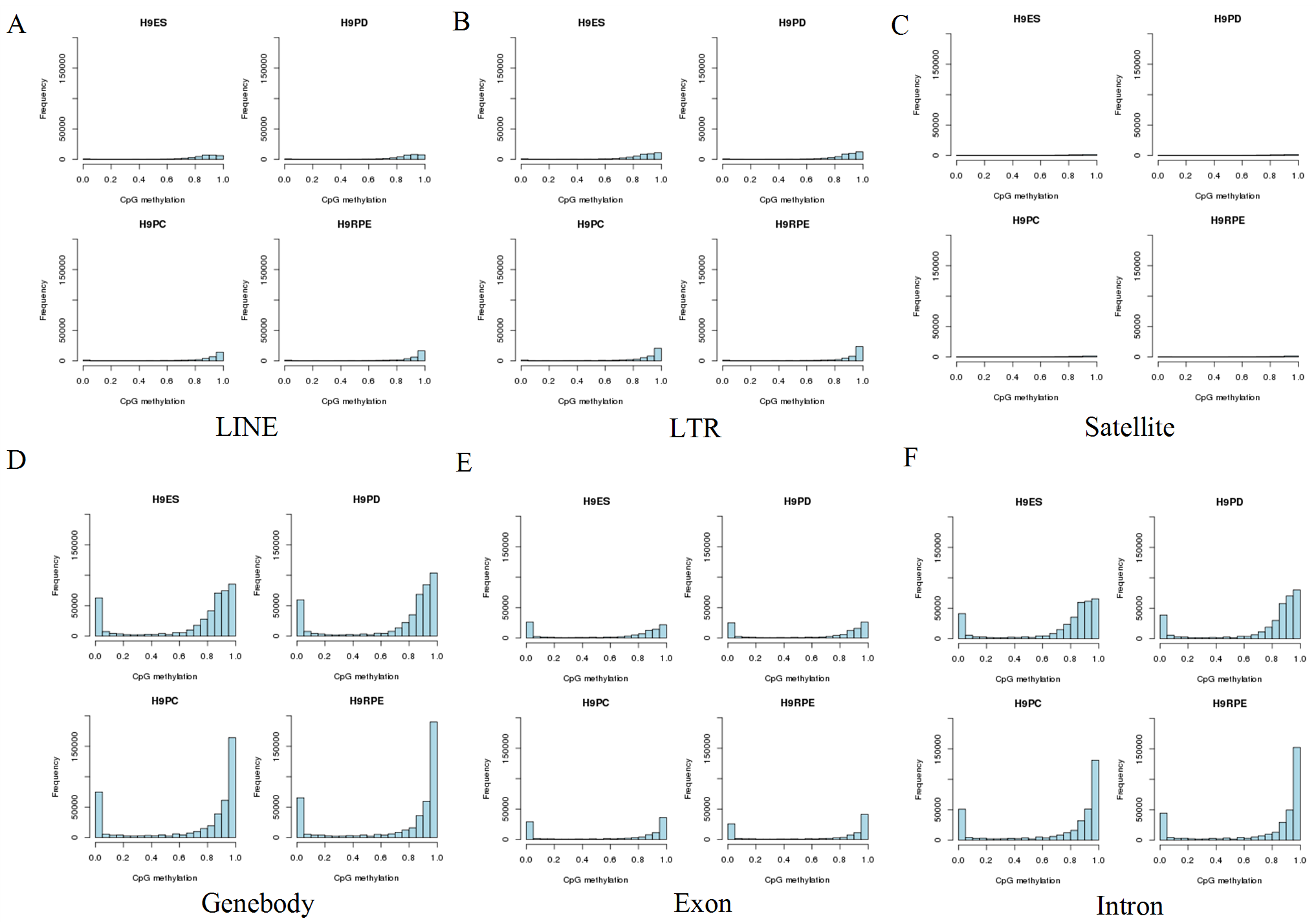

Supplement: Figure S3 — The distribution of CG methylation level for (A) LINE, (B) LTR, (C) SINE, (D) Satellite, (E) CGI, (F) Genebody, (G) Exon and (H) Intron during RPE differentiation. (TIF) [file pone.0091416.s003.tif]

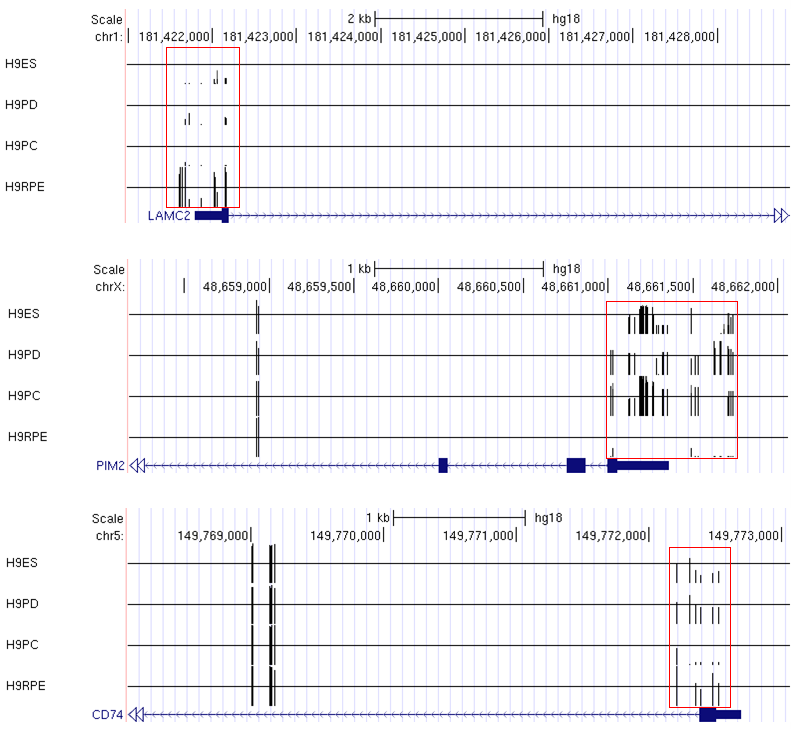

Supplement: Figure S4 — Genome browser views of DNA methylation profiles found in LAMC2, ALX3, and SALL4 genes during the course of RPE differentiation. (TIF) [file pone.0091416.s004.tif]

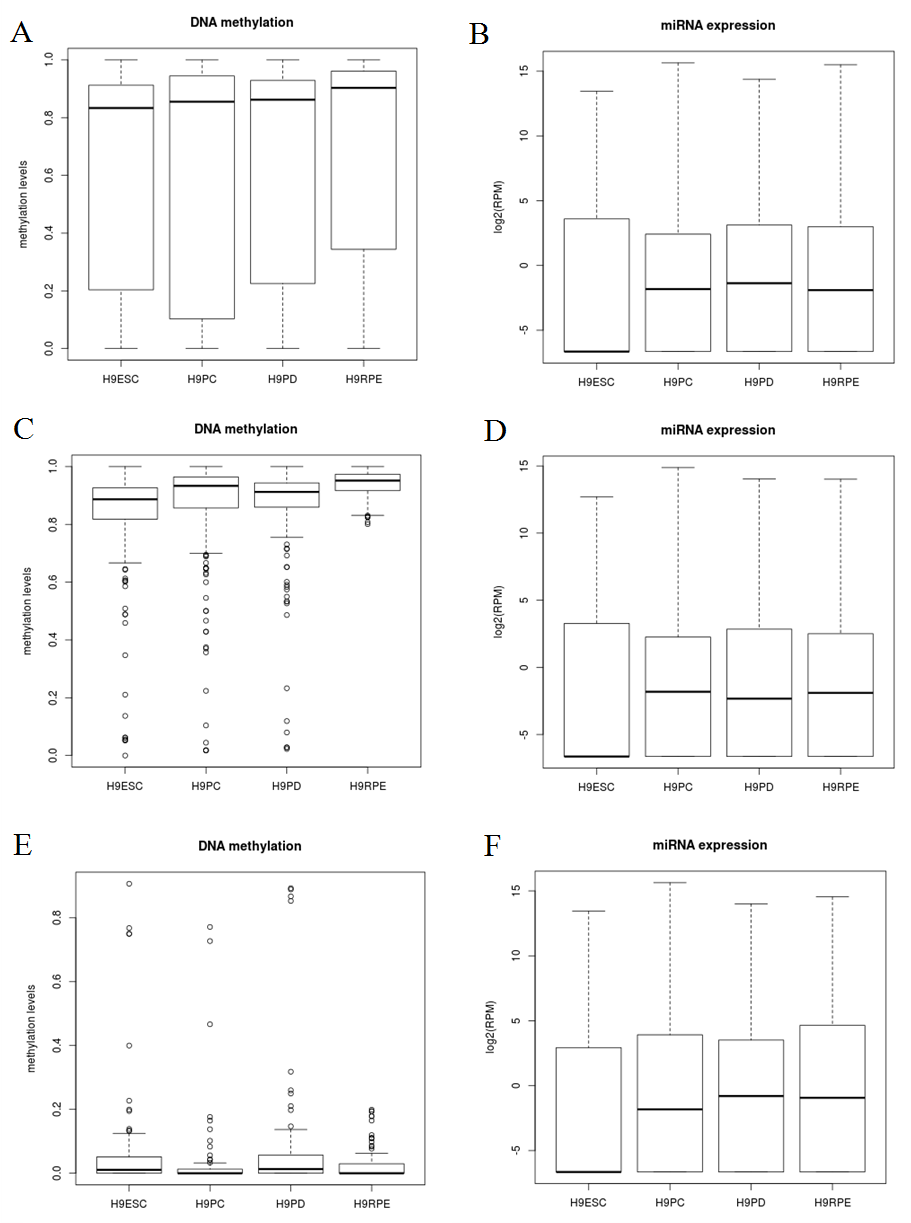

Supplement: Figure S5 — The overview of DNA methylation and expression of miRNAs. (A, C, E) Boxplots of DNA methylation levels for 419 all, 216 high and 92 low selected miRNAs, respectively. (B, D, F) Boxplots of miRNA expression levels on log scale for 419 all, selected 216 high and 92 low methylated miRNAs. (TIF) [file pone.0091416.s005.tif]
