# Supplementary material for: Integrated Analysis of DNA Methylation and RNA Transcriptome during In Vitro Differentiation of Human Pluripotent Stem Cells into Retinal Pigment Epithelial Cells
Source: PLoS One. 2014 Mar 17;9(3):e91416. doi: 10.1371/journal.pone.0091416 (PMC3956675; doi:10.1371/journal.pone.0091416)
Supplement: Table S1 — Summary of RRBS reads and mapping data. (DOC) [file pone.0091416.s006.doc]

Table S1

| **Sample** | **Cell types** | **Total reads** | **Mapped reads** | **% mapped** | **Bisulfite conversion rate (%)** | **#CG** | **#CHG** | **#CHH** | **% CG** | **% CHG** | **% CHH** | **% mC** |
| --- | --- | --- | --- | --- | --- | --- | --- | --- | --- | --- | --- | --- |
| H9 ESC | ESC | 25,040,643 | 12,991,233 | 52% | 99.02% | 1,344,086 | 1,789,407 | 4,075,699 | 67.79% | 2.68% | 0.49% | 13.58% |
| UCLA4 ESC | ESC | 24,463,989 | 11,301,627 | 46% | 99.26% | 1,478,439 | 1,963,855 | 4,468,456 | 67.60% | 1.64% | 0.30% | 13.21% |
| hiPS2 | iPSC | 23,231,842 | 11,494,563 | 49% | 99.18% | 1,512,105 | 2,025,982 | 4,673,369 | 66.08% | 1.90% | 0.33% | 12.82% |
| HDF iPSC | iPSC | 19,782,208 | 9,864,424 | 50% | 99.76% | 966,919 | 1,255,405 | 2,799,717 | 73.46% | 0.82% | 0.17% | 14.44% |
| H9 PD | Partially differentiated | 27,251,938 | 13,408,717 | 49% | 98.15% | 1,286,860 | 1,687,765 | 3,796,030 | 69.84% | 2.95% | 0.61% | 14.35% |
| H9 PC | Pigment cluster | 25,104,376 | 12,460,867 | 50% | 99.95% | 996,574 | 1,282,920 | 2,816,267 | 68.73% | 0.14% | 0.06% | 13.51% |
| H9 RPE^*^ | ESC-RPE | 25,425,997 | 12,788,225 | 50% | 99.98% | 1,170,223 | 1,520,250 | 3,395,508 | 68.08% | 0.13% | 0.07% | 13.16% |
| H9 RPE^*^ | ESC-RPE | 21,937,156 | 11,399,481 | 52% | 99.86% | 1,137,830 | 1,503,084 | 3,444,310 | 73.93% | 0.22% | 0.32% | 14.06% |
| UCLA4 RPE | ESC-RPE | 28,921,146 | 14,193,375 | 49% | 99.86% | 1,511,287 | 1,953,000 | 4,405,214 | 66.27% | 0.22% | 0.32% | 12.96% |
| hiPSC2 RPE | iPSC-RPE | 25,144,438 | 12,853,310 | 51% | 99.86% | 1,174,746 | 1,478,686 | 3,275,101 | 63.37% | 0.21% | 0.29% | 12.77% |
| HDF RPE | iPSC-RPE | 30,894,047 | 15,256,470 | 49% | 99.96% | 1,190,687 | 1,532,700 | 3,388,785 | 68.72% | 0.15% | 0.09% | 13.48% |
| fRPE1 | Fetal RPE | 18,402,412 | 9,071,527 | 49% | 99.88% | 825,776 | 1,044,556 | 2,306,533 | 66.66% | 0.26% | 0.35% | 13.44% |
| fRPE2 | Fetal RPE | 28,451,200 | 14,544,878 | 51% | 99.94% | 1,165,531 | 1,508,791 | 3,346,038 | 67.65% | 0.19% | 0.22% | 13.26% |

#CG, #CHG, and #CHH: Number of unique CpGs, CHGs and CHHs, respectively;

%CG, %CHG, %CHH, %mC: Methylation levels for CG, CHG, CHH and mC, respectively;

^*^These are technical replicates.
