# Supplementary material for: Integrated Analysis of DNA Methylation and RNA Transcriptome during In Vitro Differentiation of Human Pluripotent Stem Cells into Retinal Pigment Epithelial Cells
Source: PLoS One. 2014 Mar 17;9(3):e91416. doi: 10.1371/journal.pone.0091416 (PMC3956675; doi:10.1371/journal.pone.0091416)
Supplement: Table S2 — GO analysis via DAVID software for set of demethylated genes from PD into PC cells. (DOC) [file pone.0091416.s007.doc]

Table S2

| **Term** | **Count** | **P-Value** | **Genes** |
| --- | --- | --- | --- |
| Perinuclear region of cytoplasm | 5 | 0.014427374 | PRKCZ, MMP23B, CMYA5, RASIP1, LAMC2 |
| Regulation of cellular localization | 4 | 0.018744363 | CPLX3, ADORA2B, CRYAB, RPH3AL |
| Protein complex biogenesis | 5 | 0.027182773 | MED7, PRKCZ, HSP90AA1, CRYAB, CD74 |
| Protein complex assembly | 5 | 0.027182773 | MED7, PRKCZ, HSP90AA1, CRYAB, CD74 |
| Regulation of system process | 4 | 0.033103169 | CPLX3, PRKCZ, ARC, ADORA2B |
| Regulation of synaptic transmission | 3 | 0.03857947 | CPLX3, PRKCZ, ARC |
| Regulation of transmission of nerve impulse | 3 | 0.04440624 | CPLX3, PRKCZ, ARC |
| Plasma membrane | 19 | 0.045592631 | SETDB1, FXYD1, CPLX3, PRKCZ, ARC, ADORA2B, CMKLR1, GPER, GPR75, CRYAB, AK1, NFAM1, CD74, ESYT3, RAET1L, NRN1L, LAMC2, INPP5E, PLA2G3 |
| Regulation of neurological system process | 3 | 0.04771482 | CPLX3, PRKCZ, ARC |
