# Supplementary material for: Integrated Analysis of DNA Methylation and RNA Transcriptome during In Vitro Differentiation of Human Pluripotent Stem Cells into Retinal Pigment Epithelial Cells
Source: PLoS One. 2014 Mar 17;9(3):e91416. doi: 10.1371/journal.pone.0091416 (PMC3956675; doi:10.1371/journal.pone.0091416)
Supplement: Table S3 — GO analysis via DAVID software for set of demethylated genes from PC into RPE cells. (DOC) [file pone.0091416.s008.doc]

Table S3

| **Term** | **Count** | **P-Value** | **Genes** |
| --- | --- | --- | --- |
| Non-membrane-bounded organelle | 6 | 0.01111079 | ZNF506, ATG4A, PSMD10, MRPS21, MID2, HIST1H3I |
| Intracellular non-membrane-bounded organelle | 6 | 0.01111079 | ZNF506, ATG4A, PSMD10, MRPS21, MID2, HIST1H3I |
| Microtubule associated complex | 2 | 0.062101445 | ATG4A, MID2 |
