# Supplementary material for: Integrated Analysis of DNA Methylation and RNA Transcriptome during In Vitro Differentiation of Human Pluripotent Stem Cells into Retinal Pigment Epithelial Cells
Source: PLoS One. 2014 Mar 17;9(3):e91416. doi: 10.1371/journal.pone.0091416 (PMC3956675; doi:10.1371/journal.pone.0091416)
Supplement: Table S4 — GO analysis via DAVID software for set of remethylated genes from PC to mature RPE. (DOC) [file pone.0091416.s009.doc]

Table S4

| **Term** | **Count** | **P-Value** | **Genes** |
| --- | --- | --- | --- |
| Regulation of transcription, DNA-dependent | 22 | 6.88E-04 | RFX8, MYF6, TBX15, FOXL1, CRABP2, VSX2, WT1, YBX2, HIC1, MED7, SALL4, TCEA3, ZNF300, CSRNP1, ZNF708, OVOL1, TDGF1, ALX3, LBX2, ZNF606, PITX1, F2R |
| Regulation of RNA metabolic process | 22 | 9.23E-04 | RFX8, MYF6, TBX15, FOXL1, CRABP2, VSX2, WT1, YBX2, HIC1, MED7, SALL4, TCEA3, ZNF300, CSRNP1, ZNF708, OVOL1, TDGF1, ALX3, LBX2, ZNF606, PITX1, F2R |
| Limb morphogenesis | 5 | 0.002426948 | SALL4, CRABP2, GREM1, ALX3, PITX1 |
| Appendage morphogenesis | 5 | 0.002426948 | SALL4, CRABP2, GREM1, ALX3, PITX1 |
| Limb development | 5 | 0.002802151 | SALL4, CRABP2, GREM1, ALX3, PITX1 |
| Appendage development | 5 | 0.002802151 | SALL4, CRABP2, GREM1, ALX3, PITX1 |
| Zinc ion binding | 25 | 0.003615112 | PRKCZ, AEBP1, UQCRC1, WT1, RNF212, HIC1, TCEA3, ZNF300, ZNF708, OVOL1, MT1E, MT1H, ZNF606, SETDB1, MT1L, BHMT2, ZBTB7C, UPB1, RPH3AL, MMP15, ZFR, RNF112, CBLC, SALL4, MMP23B, USP44 |
| Regulation of transcription | 26 | 0.003927324 | AEBP1, CRABP2, WT1, YBX2, HIC1, TCEA3, ZNF300, ZNF708, OVOL1, TDGF1, ZNF606, ALX3, PITX1, RFX8, MYF6, SETDB1, FOXL1, TBX15, NFAM1, VSX2, MED7, SALL4, CSRNP1, KLHL31, LBX2, F2R |
| Cell surface | 8 | 0.004562867 | LY75, TDGF1, PCSK9, NFAM1, KIT, MMP15, GREM1, CD74 |
| Striated muscle tissue development | 5 | 0.004700616 | MYF6, ACTC1, TDGF1, PITX1, F2R |
