# Supplementary material for: Integrated Analysis of DNA Methylation and RNA Transcriptome during In Vitro Differentiation of Human Pluripotent Stem Cells into Retinal Pigment Epithelial Cells
Source: PLoS One. 2014 Mar 17;9(3):e91416. doi: 10.1371/journal.pone.0091416 (PMC3956675; doi:10.1371/journal.pone.0091416)
Supplement: Table S5 — GO analysis via DAVID software for fRPE-specific demethylated genes comparing with hESC-RPEs and iPSC-RPEs. (DOC) [file pone.0091416.s010.doc]

Table S5

| **Term** | **Count** | **P-Value** | **Genes** |
| --- | --- | --- | --- |
| Homophilic cell adhesion | 6 | 2.03E-05 | PCDHGA11, PCDHB18, PCDHB4, PCDHA1, PCDHGA5, PCDHGB5 |
| Chromatin assembly or disassembly | 5 | 3.10E-04 | HIST1H2BM, HIST1H3A, HIST1H2AJ, NAP1L2, KAT5 |
| Cell-cell adhesion | 6 | 6.70E-04 | PCDHGA11, PCDHB18, PCDHB4, PCDHA1, PCDHGA5, PCDHGB5 |
| Nucleosome assembly | 4 | 0.001309639 | HIST1H2BM, HIST1H3A, HIST1H2AJ, NAP1L2 |
| Chromatin assembly | 4 | 0.001449155 | HIST1H2BM, HIST1H3A, HIST1H2AJ, NAP1L2 |
| Protein-DNA complex assembly | 4 | 0.001649238 | HIST1H2BM, HIST1H3A, HIST1H2AJ, NAP1L2 |
| Nucleosome organization | 4 | 0.001755446 | HIST1H2BM, HIST1H3A, HIST1H2AJ, NAP1L2 |
| Chromatin organization | 6 | 0.002699903 | HIST1H2BM, BRCC3, HIST1H3A, HIST1H2AJ, NAP1L2, KAT5 |
| Calcium ion binding | 8 | 0.002761379 | PCDHGA11, PCDHB18, PCDHB4, CABP7, PCDHA1, PCDHGA5, TPM4, PCDHGB5 |
| DNA packaging | 4 | 0.003373058 | HIST1H2BM, HIST1H3A, HIST1H2AJ, NAP1L2 |
